# CasCollect: targeted assembly of CRISPR-associated operons from high-throughput sequencing data

**DOI:** 10.1093/nargab/lqaa063

**Published:** 2020-09-03

**Authors:** Joshua D Podlevsky, Corey M Hudson, Jerilyn A Timlin, Kelly P Williams

**Affiliations:** Molecular and Microbiology, Sandia National Laboratories, Albuquerque, NM 87185, USA; Computational Biology and Biophysics, Sandia National Laboratories, Albuquerque, NM 87185, USA; Computational Biology and Biophysics, Sandia National Laboratories, Albuquerque, NM 87185, USA; Systems Biology, Sandia National Laboratories, Livermore, CA 94550, USA

## Abstract

CRISPR arrays and CRISPR-associated (Cas) proteins comprise a widespread adaptive immune system in bacteria and archaea. These systems function as a defense against exogenous parasitic mobile genetic elements that include bacteriophages, plasmids and foreign nucleic acids. With the continuous spread of antibiotic resistance, knowledge of pathogen susceptibility to bacteriophage therapy is becoming more critical. Additionally, gene-editing applications would benefit from the discovery of new *cas* genes with favorable properties. While next-generation sequencing has produced staggering quantities of data, transitioning from raw sequencing reads to the identification of CRISPR/Cas systems has remained challenging. This is especially true for metagenomic data, which has the highest potential for identifying novel *cas* genes. We report a comprehensive computational pipeline, CasCollect, for the targeted assembly and annotation of *cas* genes and CRISPR arrays—even isolated arrays—from raw sequencing reads. Benchmarking our targeted assembly pipeline demonstrates significantly improved timing by almost two orders of magnitude compared with conventional assembly and annotation, while retaining the ability to detect CRISPR arrays and *cas* genes. CasCollect is a highly versatile pipeline and can be used for targeted assembly of any specialty gene set, reconfigurable for user provided Hidden Markov Models and/or reference nucleotide sequences.

## INTRODUCTION

Clustered regularly interspaced short palindromic repeats (CRISPR) arrays and the CRISPR associated (Cas) proteins constitute adaptive immune systems that bacteria and archaea use to defend against invading plasmids, bacteriophage viruses and other foreign DNAs or RNAs (1,2). The effector of a CRISPR/Cas immune system is a ribonucleoprotein (RNP) complex, composed of one or more catalytic Cas proteins and a CRISPR RNA that targets foreign nucleic acids for endonucleolytic cleavage. CRISPR/Cas systems are present in many bacteria and archaea and have been classified into two classes, six types and 22 subtypes based on the composition of *cas* genes within each system (3).

The first CRISPR/Cas effector developed for biotechnology, remaining the focus for gene-editing applications, was the simple Class 2 type II CRISPR/Cas system. This system requires the CRISPR RNA, the *trans*-activating RNA, and the nucleolytic Cas9 protein for targeted DNA cleavage (4). The CRISPR and *trans*-activating RNA were further simplified into an engineered single guide RNA. However, this Cas9 system was developed from human pathogens and has been reported to invoke an immune response (5). Although Cas9 remains the most popular system for biotechnology applications, there is ongoing work to develop Class 2 type V CRISPR/Cas systems—that include Cas12 (Cpf1)—as well as searches for other new systems. Additionally, Class 2 type VI CRISPR/Cas systems that target RNA, Cas13 (C2c2), are of interest for transcriptional modification in-place-of gene-editing applications. The need for discovering CRISPR arrays and Cas proteins is furthered by multi-drug-resistant infections and the potential of bacteriophage therapeutics (6). Tools that improve the efficacy and speed of CRISPR/Cas discovery will be useful for evaluating whether a bacterial pathogen is susceptible to bacteriophage therapy.

The discovery of novel CRISPR/Cas systems from non-pathogenic organisms is essential for identifying systems for therapeutic gene-editing, while rapid detection of these systems from pathogenic organisms is critical for applying the most appropriate and effective phage therapy. There have been progressive improvements in the identification and annotation of CRISPR/Cas system components from individual genomes and from previously assembled metagenomes (7–12) with an array of tools available (13–16). More recent identification of *cas* genes have employed Hidden Markov Models (HMM) and Position Specific Scoring Matrices (PSSM) that enable deep homology searches for novel members of known *cas* gene families (8–10). HMMs and PSSMs have been applied toward annotating well-defined and assembled bacterial and archaeal genomes. The rate of discovering CRISPR/Cas system components could be improved significantly by avoiding the complete assembly of the raw sequencing read datasets, instead identifying reads that match *cas* genes and/or CRISPR arrays and using these ‘seed’ reads to prime the targeted assembly of only the regions of the genome corresponding to CRISPR/Cas systems.

Herein we report a software pipeline, CasCollect, for the targeted assembly and annotation of *cas* genes and CRISPR arrays from raw sequencing datasets. We demonstrate the utility of CasCollect with unassembled high-throughput sequencing reads from bacterial isolate genomes, simulated metagenome and available actual metagenome datasets. Evaluating CasCollect with a simulated metagenomic dataset, we successfully identified all known *cas* genes, accurately assembling and annotating the *cas* operons. Our CasCollect pipeline reduced the timing for *cas* genes identification and annotation from unassembled metagenomic sequencing data by almost two orders of magnitude compared with the complete assembly. As a case study of CasCollect for determining phage therapy potential, we successfully identified the CRISPR arrays and *cas* operons from publicly available patient data for 66 antibiotic resistant *Pseudomonas aeruginosa* clinical isolates—nearly all had only unassembled sequencing read data available. CasCollect is a versatile software tool for the identification and annotation of CRISPR arrays and *cas* operons from unassembled high-throughput sequencing datasets. The pipeline software is flexible, capable of the targeted assembly of other specialty gene sets when supplied with relevant HMM collections and/or reference DNA sequences.

## MATERIALS AND METHODS

### Data sources

The genomic and metagenomic datasets used in this study were downloaded from the NCBI downloaded from the Sequence Read Archive (SRA) at (http://www.ncbi.nlm.nih.gov/sra). Isolate genome datasets were *Escherichia coli* KLY isolate (SRR1424625), *P. aeruginosa* VA-134 isolate (SRR2939129) and *Streptococcus pyogenes* M39 isolate (SRR5280756). For simulating a metagenomic dataset, these three isolates were combined with mouse sequencing data (SRR1752459) to increase sample complexity. For the metagenomic study, we used the following datasets for ground water, deep water biosphere (SRR10598175); Lake Redon in Central Pyrenees, Spain (ERR472738); Artic permafrost (SRR11195315); and peatland wetlands (SRR5823773). Unassembled read datasets of phage therapy candidates were for 66 antibiotic resistant *P. aeruginosa* isolates that are distributed by the CDC & FDA Antibiotic Resistance Isolate Bank. All sequencing data were from Illumina sequencing platforms and downloaded as SRA files with Fastq files extracted by executing the SRA toolkit command fastq-dump with paired-end files split (17).

### CasCollect development and targeted gene assembly

CasCollect was developed in Python and Perl languages with the pipeline publicly available for download under the terms of the GNU General Public License version 3 at https://github.com/sandialabs/CasCollect. Installation requirements and documentation are provided in the download. A check script for dependencies will download and extract missing software. All tests reported for this work were performed on a system setup with 100 Intel Xeon CPUs at 2.40GHz and 2 Tb RAM. CasCollect was designed for a POSIX-compliant operating system that include Unix and Linux distributions. CasCollect dependencies are BBTools 38.84 (https://jgi.doe.gov/data-and-tools/bbtools/), Seqtk (https://github.com/lh3/seqtk), FragGeneScanPlus (FGS+, https://github.com/hallamlab/FragGeneScanPlus), HMMER v3.3 (http://hmmer.org/), VSEARCH (https://github.com/torognes/vsearch), SPAdes 3.14.1 (http://cab.spbu.ru/software/spades/) and CRISPRCasFinder (https://crisprcas.i2bc.paris-saclay.fr/). CRISPRCasFinder was parallelized through a Perl script for the number of CPUs defined by the user input and skips contigs below a size cutoff to generate a GFF3 output file. The CasCollect pipeline includes read filtering, seed generation, subset read expansion, assembly, and annotation for *cas* genes and CRISPR arrays.

### CasCollect parameters

**Figure 1.**
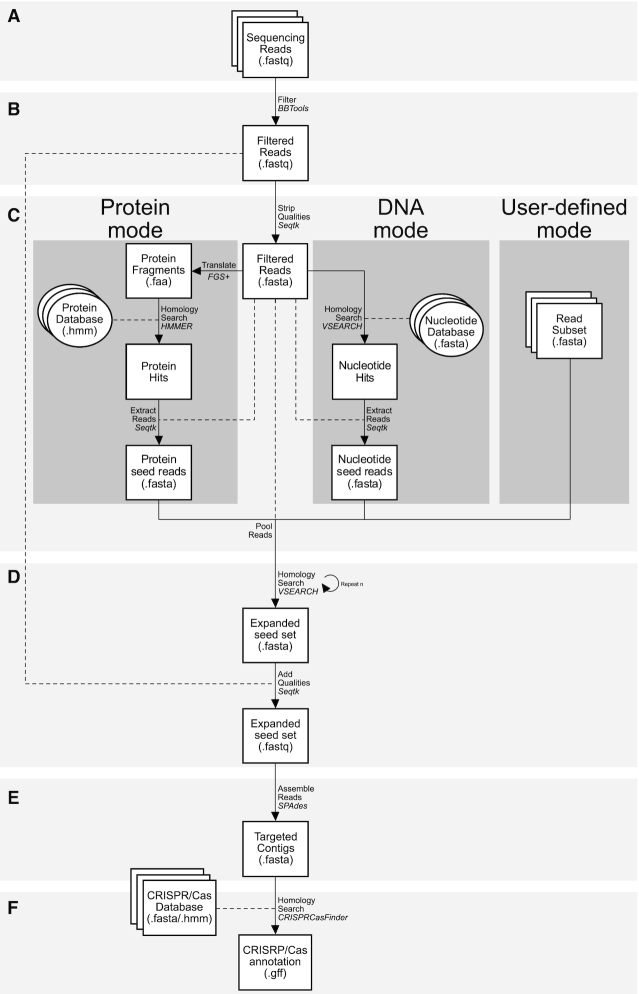
Workflow for the CasCollect pipeline. CasCollect processes an initial high-throughput sequencing read dataset (**A**) by read filtering (**B**), seed generation (**C**), read subset expansion (**D**), assembly (**E**) and annotation (**F**). (A) The sequencing read dataset requires quality scores for assess the confidence for each base call for the subsequent filtering step. (B) Read filtering can be ignored, for trimming of adapter sequences and low-quality regions, or cleaning that performs trimming and removes reads matching a reference of undesired sequence(s) that can be supplied by the user. For paired-end reads, both trimming and cleaning will merge reads with over lapping regions. (C) Seed reads can be generated by Protein mode, DNA mode and/or a user-defined read subset (dark gray boxes). Protein mode translates the reads for searching with either the built-in protein profile HMMs or a user-defined set. DNA mode searches for matches to user-defined reference sequence(s). User-defined mode allows for any subset of reads or sequences be used for seed expansion. The seed generation modes can be invoked independently or concurrently within a single run of the program. (D) The number of cycles of read subset expansion can be varied to generate larger or smaller expanded read sets. The (E) targeted assembly using this subset of reads and (F) annotation of the assembled contigs are optional for identifying *cas* genes and CRISPR arrays.

The CasCollect pipeline has several parameters that can be altered for user specific workflows, described in detail with the -h command. The short DNA sequencing read reads input can accommodate single- or paired-end sequencing data with -single [file.fastq] or -fwd [file.fastq] and -rev [file.fastq], respectively (Figure 1A). The filtering option is set to false by default and the sequencing reads will be run for the seed generation and downstream workflow (Figure 1B). Setting the flag --trim will perform adapter trimming and read merging for paired-end data. The flag --clean performs the trim function with the addition of removing sequencing reads that match a user-defined set of undesired nucleic acid sequence(s) set with -ref [file.fasta]. For seed generation, protein mode searching for *cas* genes by default using a set of 120 HMM profiles (13) included with the program (Figure 1C). The Cas protein profile HMMs can be substituted with -hmm [file.hmm] and protein mode disabled with the flag --noprot. DNA and user-defined modes are disabled by default and can be activated by the flags --nucl and --seed; the search sequences are set with -query [file.fasta] and -define [file.fasta], respectively. The number of rounds of seed expansion is defaulted to 5 and can be changed with -cycle [number], while the match is set for 95% and can be changed with -match [number] (Figure 1D). Read sequence assembly and annotation are default and can be disabled by the flags --noassembly (Figure 1E) and --noannotate (Figure 1F), respectively. The flag --meta runs metaSPAdes in-place-of the SPAdes assembly.

### Unassembled genomic DNA comparative analysis

For the *E. coli* KLY, *P. aeruginosa* VA-134 and *S. pyogenes* M39 bacteria isolates, CasCollect was run with the default parameters for a protein homology read search with the Cas HMM profiles and following parameters: --trim -cycle 2 -cpu 100 -mem 2000. The pooled simulated and metagenomic dataset was run with the following parameters: --trim --meta -cpu 100 -mem 2000. Datasets from the CDC & FDA Antibiotic Resistance Isolate Bank panel of *P. aeruginosa* isolates were run with similar parameters as the bacteria isolates: --trim -cycle 2 -cpu 100 -mem 2000 appended with --nucl -query Pseudomonas_aeruginosa_DK2.fas for DNA mode to search for isolated CRISPR arrays. The complete assembly used the CasCollect filtered and trimmed run through SPAdes with the same number of CPUs and amount of RAM as CasCollect. For the metagenomic and simulated metagenomic datasets, metaSPAdes was run in-place-of the SPAdes assembly (18).

### Progressive read collection analysis

The metagenomic dataset was run with the CasCollect pipeline with zero to five cycles of read subset expansion. Each of these read sets and the whole sequencing dataset were mapped onto the largest *cas* operon for each metagenome with bowtie2 with default parameters (19). The read coverage was extracted with SAMtools (20) using the depth command and -a parameter to output coverage for the full-length contig.

## RESULTS AND DISCUSSION

To overcome the requirement of assembling the entire set of short DNA sequencing reads prior to searching for gene(s) of interest, we developed CasCollect. CasCollect identifies short DNA sequencing reads that match HMM profiles for *cas* protein genes and collects these reads for targeted assembly. To identify CRISPR arrays isolated from *cas* genes, CasCollect can collect reads that match to CRISPR repeats. However, this approach may not necessarily identify all reads from a genomic CRISPR/Cas locus and the possibility of discontinuous coverage of reads would prevent complete assembly of the locus. To infill the potentially missing regions between profile matching reads, the seed reads are expanded to include neighboring adjacent reads. This seed expansion is performed with stringent sequence similarity matches to reduce non-specific read matching. This expanded CRISPR/Cas read set typically constitutes a very small fraction of the total sequencing reads and bypasses the computational overhead associated with whole genome reconstruction by assembling only the targeted region(s) of interest. CasCollect can perform targeted assembly of specialty gene sets other than *cas* and CRISPR genes by specifying other HMM collections and/or reference DNA sequence files.

### CasCollect pipeline

CasCollect was written as a python wrapper for various selectable modules that encompass additional programs pipelined together for use on UNIX and Linux architecture operating systems (Figure 1). The CasCollect pipeline was created from five distinct and optional steps: (i) read filtering, (ii) seed generation, (iii) read subset expansion, (iv) assembly and (v) annotation. This pipeline relies on several third-party programs that include: HMMER (21), VSEARCH (22), SPAdes (23) and CRISPRCasFinder (13) for the targeted assembly of gene(s) of interest from either single or paired end data.

The option of sequencing read filtering is performed either as a ‘trimming’ or ‘cleaning’ step by executing the two BBTools programs (https://jgi.doe.gov/data-and-tools/bbtools/) bbduk and bbmerge. The trimming and cleaning options are implemented by bbduk on the short DNA sequencing reads (Figure 1A). Trimming removes adapters from reads, while the cleaning option removes adapters and includes the removal of defined ‘contaminating’ sequences that match a user-defined Fasta file of sequences (Figure 1B). The most common use for the cleaning option is to remove off-target sequencing of undesired additional biological materials, such as human DNA in microbiome samples. For paired-end data, bbmerge is then used to identify and merge overlapping sequencing reads (24). This step is ignored for single-end sequencing data or when neither ‘trimming’ or ‘cleaning’ steps are performed.

The initial seed collection comprises three main modes: ‘Protein mode’, ‘DNA mode’ and ‘User-defined mode’ (Figure 1C). For seed collection, the sequencing reads are converted to Fasta format with quality scores stripped using Seqtk (https://github.com/lh3/seqtk). Protein mode employs FragGeneScanPlus (25) to translate the most likely of the six open reading frames for each read for homology searching by HMMER (21) with a default set of 120 Cas protein family profile HMMs (13) as query. DNA mode implements VSEARCH (22) for identity matching to a user-defined set of CRISPR array repeats for the identification of CRISPR arrays that may be found isolated from the *cas* genes. We have included 105 CRISPR repeat sequences (13) as individual Fasta files with CasCollect to aid in CRISPR array detection. The user-defined mode uses a specified set of reads as the seed set (Figure 1C). The protein, DNA, and user-defined modes can be invoked independently or concurrently within a single run of the program pipeline to search for *cas* genes, isolated CRISPR arrays, or other sequences. Thus, any combination of these three seed generation modes can be applied in a single run of CasCollect. CasCollect is flexible and capable of assembling other specialty protein or DNA gene sets by using any set of protein profile HMMs (-hmm), reference DNA sequences (--nucl and -query), or read seed sets provided the user (--seed and -define).

Read subset expansion, in both directions from each seed read, is performed by reiterative implementations of VSEARCH usearch global search function (Figure 1D). The default identity match has been set to a moderately high value of 95%, however, this can be adjusted with the -match command. The initial read subset expansion is performed with the seed reads from the DNA, protein, and/or user-defined mode(s) as the query against the entire sequencing read set as the database. The matching reads are separated from the remaining reads from the dataset and then used as the query for the subsequent VSEARCH search with the unmatched reads then used as the database for this next query. This process eliminates redundant searches by removing reads from the database set that have been matched in the previous cycle instead of the entire sequencing read set. The default number of these read subset expansion cycles is set at five and can be adjusted with the -cycle command. After the final cycle of read subset expansion, all matching reads are pooled and the sequencing quality scores are added back, generating Fastq files for assembly and contig building with quality scores.

The assembly function employs the SPAdes assembler (23) using the subset of reads from the seed and expansion steps (Figure 1E). For metagenomes, metaSPAdes is used in-place-of SPAdes. This targeted assembly is specific to the queries from the protein, DNA, and user-defined modes, by default *cas* genes and CRISPR arrays; yet any sequence can be targeted for assembly with the corresponding protein profile HMMs, reference DNA sequences, or read seed sets. By merely using a small subset of reads for targeted assembly, we avoid the time intensive process of attempting to assemble the complete genome using all the sequencing reads. This targeted assembly reduces computational resources and time, bypassing difficult assembly regions outside the regions of interest.

The final step in the pipeline is the identification and annotation of *cas* genes and CRISPR arrays from the assembled contig sequences (Figure 1F). The targeted assembly contigs are searched with the CRISPRCasFinder program (13). The annotation of a smaller targeted assembly further reduces computational resources and time, bypassing searching most of the genome. Identification and annotation are parallelized with a Perl script, which additionally collates results into a GFF3 file for describing the CRISPR/Cas systems, genes, and arrays present in the in the assembled contig sequences.

### Bacterial isolates

We initially benchmarked the timing and quality of our CasCollect pipeline with sequencing read datasets corresponding to well-annotated bacterial isolate genome assemblies. The complete and annotated genomes allowed for definitive checks to validate targeted assembly qualities and automated annotation. The CasCollect pipeline was compared against conventional full genome assembly using SPAdes for the entire sequencing read set, herein referred to as ‘complete assembly’ (Supplemental Figure S1). For this comparison, we tested publicly available short paired-end genomic DNA sequencing reads from isolates of *E. coli* strain KLY, *P. aeruginosa* strain VA-134 and *S. pyogenes* strain M39 that had read lengths of 90, 100 and 226 bp, respectively. For our comparative analysis, we ran the CasCollect pipeline and a complete assembly pipeline as the baseline. The complete assembly pipeline is highly similar to CasCollect with identical read filtering and annotation steps (Supplemental Figure S1A). The difference between the pipelines was the omission of the seed generation and expansion steps that influence the reads used for the assembly by SPAdes. The complete assembly pipeline used all the filtered reads for the SPAdes assembly step, while CasCollect generated instead of a much smaller read subset. This pipeline comparison maintained identical conditions for the sequencing read filtering and the annotation of the assembled sequences between CasCollect and the complete assembly. Both CasCollect and complete assembly pipelines were able to identify all known previously identified *cas* genes within each of the three bacterial isolate genomes.

The timing for the *E. coli* strain KLY, *P. aeruginosa* strain VA-134 and *S. pyogenes* strain M39 bacterial isolates demonstrated relatively similar timings for the CasCollect and complete assembly pipelines, with timings of 23.3/17.4, 51.2/33.3 and 21.6/31.5 min (Supplemental Figure S1B). The most time intensive step for CasCollect was the seed generation and expansion, while the assembly and annotation were relatively fast (Supplemental Figure S1C). In contrast, the assembly step for the complete assembly pipeline was the overwhelming contributor for the program runtime. For the *E. coli* strain KLY dataset, the complete assembly required more time for annotation, which was likely due to the number of contigs generated—146 contigs for the complete assembly compared with 19 from CasCollect (Supplemental Figure S1D). In addition to more contigs, the length of the contigs was far greater with 28 exceeding 50 Kb (Supplemental Figure S1E). For *P. aeruginosa* strain VA-134 the number of assembled contigs and the overall timings were similar for CasCollect and the complete assembly pipelines at 111 and 162, respectively (Supplemental Figure S1D). Interestingly, CasCollect generated more contigs for *S. pyogenes* strain M39 than the complete assembly at 89 and 44, respectively. This is explained by the complete assembly generating fewer, yet larger contigs (Supplemental Figure S1E).

An alternative software pipeline for CasCollect was built using PRICE (26) to replace the read subset expansion and assembly (Supplemental Figure S1A). The PRICE pipeline is highly similar to CasCollect with identical read filtering, seed generation and annotation steps. The difference between the pipelines was the read subset expansion and assembly. The PRICE pipeline used the seed generation reads for iterative extension to build the contigs (Supplemental Figure S1A). The pipeline comparison maintained identical conditions for the sequencing read filtering and the annotation of the assembled sequences between CasCollect and PRICE. Both CasCollect and PRICE pipelines were able to identify all known previously identified *cas* genes within each of the three bacterial isolate genomes. The timings for the PRICE pipeline for *P. aeruginosa* strain VA-134 and *S. pyogenes* strain M39 datasets were highly similar to CasCollect at 51.2/50.1 and 21.6/22.6 min, respectively (Supplemental Figure S1B). For the *E. coli* strain KLY, the PRICE pipeline had difficulties with generating the contig spanning the *cas* operon. While the *P. aeruginosa* strain VA-134 and *S. pyogenes* strain M39 datasets were successful with contig generation with five cycles, the *E. coli* strain KLY dataset required 100 cycles. This is reflected in the massive timing increase at over 2 h and 123.5 min. The more than 5-fold time increase was likely due to the read length, with the *E. coli* strain KLY dataset having the shortest DNA sequencing reads. Using short sequencing reads as seed contigs for PRICE is far from the intended function of PRICE.

### Simulated metagenome

To estimate the CasCollect pipeline performance with a metagenomic DNA sequencing dataset, we benchmarked the three pipelines with a simulated metagenomic dataset (Supplemental Figure S2). For the simulated metagenomic data, we pooled together the *E. coli* KLY, *P. aeruginosa* VA-134 and *S. pyogenes* M39 bacterial isolate genome sequencing reads. Mouse sequencing reads were spiked in to increase the read complexity. This created a curated metagenomic dataset, with known CRISPR/Cas systems for evaluating the quality of the metagenomic assembly for *cas* gene operons within a complex of several genomes. The simulated metagenome read dataset did not affect the quality of the assembly or annotation for CRISPR/Cas systems for either CasCollect or the complete assembly pipelines. However, the PRICE pipeline was unable to assemble contigs spanning the complete *cas* operon for *E. coli* KLY or *S. pyogenes* M39. Increasing the cycle number to 200 built a contig to span the complete *cas* operon for *E. coli* KLY. Yet even increasing the cycle number to 500, PRICE was unable to build a contig to span the *S. pyogenes* M39 *cas* operon. Increasing the cycle number to 500 dramatically increased the timing for the PRICE pipeline to almost 90 h (Supplemental Figure S2A and B).

CasCollect reduced the run time modestly by 36% relative to the complete assembly (Supplemental Figure S2B). The CasCollect runtime was 1.6 h compared with the complete runtime of 2.5 h. Compared with PRICE that failed to assemble all *cas* operons, CasCollect and the complete assembly pipelines were almost 57- and 37-fold faster and completed building all *cas* operons. As was previously seen with the isolate genomes, the vast majority of the run time for CasCollect was used on the seed generation and expansion steps. With fewer reads, CasCollect required less time for contig assembly compared with the complete assembly pipeline and this was reflected as a smaller percentage of the overall run time (Supplemental Figure S2C). Annotation was faster with CasCollect with merely 723 contigs compared with the 10 467 contigs for the complete assembly (Supplemental Figure S2D). PRICE generated merely 56 contigs. Analysis of the contig size populations found CasCollect generated no contigs larger than 50 Kb (Supplemental Figure S2E). In sharp contrast, the complete assembly pipeline generated 69 contigs larger than 50 Kb. For PRICE, 26 of the 56 contigs were larger than 50 Kb. Our simulated metagenomic data analysis demonstrated that CasCollect targeted assembly is more efficient for the assembly of genes of interest and avoided excessive assembly by an order of magnitude for our simulated metagenome.

### Metagenome

**Figure 2.**
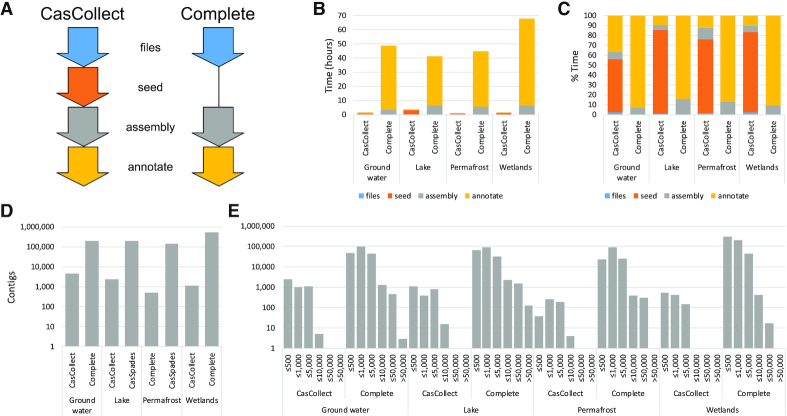
Comparative analysis of the CasCollect and complete assembly pipelines for metagenomic datasets. (**A**) Schematic of the four steps for the CasCollect and complete assembly pipelines: files (blue) for trimming and stripping qualities; seed (orange) for the seed generation, expansion and reassigning qualities; assembly (gray) for contig building; and annotate (yellow) for detecting *cas* genes and CRISPR arrays to generate a GFF3 output file. (**B**) Timings for each pipeline for each metagenome and steps. (**C**) Percentage breakdown for time required for each step. (**D**) Number of contigs generated by each pipeline with (**E**) the length distribution for CasCollect and complete assembly. Colors for each step in (A) are used for the charts in (B) and (C).

The analysis of the simulated data indicated that our CasCollect pipeline accurately assembled and annotated *cas* gene operons, while improving speed compared with the complete assembly pipeline. We next investigated how CasCollect would perform with actual, instead of simulated, metagenomic datasets. For this, we analyzed four metagenomic dataset from a variety of sources (Figure 2). We performed a similar comparative analysis as previously with CasCollect and complete assembly pipeline as the baseline (Figure 2A). The complete assembly pipeline used the entire filtered read dataset as input for assembly, while CasCollect used a subset of these reads. Otherwise the two pipelines were identical for the initial read processing and downstream annotation. Our CasCollect pipeline had a dramatic improvement for the overall run time compared with the complete assembly pipeline (Figure 2B). On average for the tested metagenomes, CasCollect required merely 3% of the time required for the complete assembly; almost two orders of magnitude improvement at 1.7 ± 0.3 h and 50.6 ± 3.0 h, respectively. CasCollect reduced the over two days of runtime for the complete assembly to <2 h. This massive improvement for the timings of the actual metagenomic data was likely due to the greater complexity of numerous genomes, while our simulated metagenomic data comprised four genomes. As previously observed for the isolate genomes and pooled simulated metagenomic datasets, the vast majority of the timing for CasCollect was for the seed generation and expansion at an average of 73.7 ± 3.4% the total run time (Figure 2C). For the compete assembly, the average time for the read assembly alone was 5.5 ± 0.3 h; 3-fold the total time for the entire CasCollect pipeline. The longer timing for the complete assembly pipeline was exacerbated by the timing for the annotation that was on average 45.1 ± 2.9 h and comprised 88.7 ± 0.9% of the total run time for the complete pipeline. We also attempted to perform analysis of these four metagenomes with our PRICE pipeline (Supplemental Figure S3). Running the PRICE pipeline for 50 cycles had an average runtime of 5.5 ± 0.6 h. This was more 3-fold the timing for CasCollect, yet, failed to generate any contigs that spanned the entire *cas* operon for any of the four metagenomes. Thus, PRICE does not appear to function well for our targeted assembly, likely due to the short DNA sequencing read lengths used as seeds for building the contigs.

To investigate the cause of the large run time discrepancy for the two pipelines, we characterized the contig number and length distributions (Figure 2D and E). CasCollect generated on average two orders of magnitude fewer contigs than the complete assembly at 2.1 ± 0.5K and 269.9 ± 4.7K, respectively (Figure 2D). Analysis of the contig size distribution revealed that none of the CasCollect runs for the metagenomes generated a contig larger than 50 Kb and only one contig larger than 10 Kb (Figure 2E). The contigs for the complete assembly were far greater in size with an average of 33 contigs greater than 50 Kb. The >100-fold additional contigs and longer lengths for the complete assembly are likely the cause for the far greater annotation analysis run time. Our Perl script for automating the annotation had set a size cutoff of 500 bp, which reduced the total number of contigs analyzed for the complete assembly to an average of 161K contigs. CasCollect generated an average of 1.1K contigs greater than 500 bp, still >100-fold additional contigs for the complete assembly pipeline to analyze. This demonstrates that the almost two orders of magnitude improved timing for the CasCollect was due to the targeted assembly reducing the time for the assembly and the reduced number of contigs for analysis.

**Figure 3.**
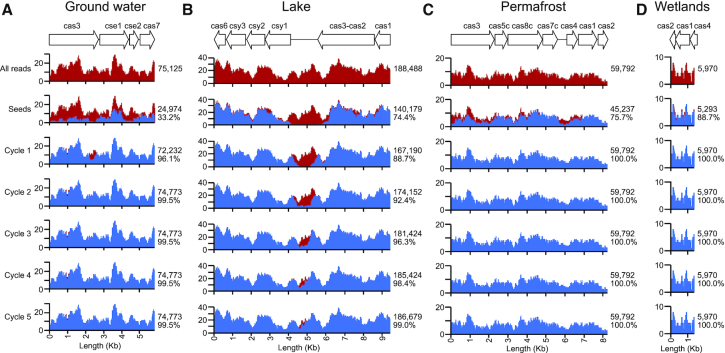
Analysis for the completeness of read collection by CasCollect. Read coverage analysis per each reiterative cycle of read subset expansion for the largest *cas* operons found in each metagenome dataset (**A**–**D**). (Top) A schematic for the *cas* genes within each operon drawn to scale above the overlay of all mappable reads and reads collected at each cycle of subset read expansion. All reads (red) denotes all mappable reads from each metagenome sequencing dataset. Seeds (blue) is the initial seed set expanded by cycles of read subset expansion (Cycle #, blue). (Right) is the number of reads from each step in CasCollect and for Seeds and Cycle # the percent of all mappable reads.

Read coverage across the *cas* genes and the intervening sequences is critical for the assembly of *cas* gene operons. Low sequencing read coverage and gapped regions lacking sequencing read coverage due to the incomplete collection of reads from the metagenomic datasets would result in the premature termination of contig building, resulting in fragmented *cas* genes and operons. To determine the completeness of the read collection for CasCollect, we analyzed read coverage for each cycle of read subset expansion for the largest *cas* operon from each of the four metagenomes (Figure 3). The reads were extracted for the seeds and each cycle of seed subset expansion. These reads as well as the total reads were then mapped onto the *cas* operons. For the ground water *cas* operon, the initial seed generation did well for continuous coverage with a few short regions lacking any reads (Figure 3A). The average coverage was 33.2% of the total mappable reads. After a single cycle of subset read expansion, this increased to 96.1% and read coverage had no gaps across the *cas* operon. After the second cycle, the average read coverage was 99.5% and remained unchanged for the following three cycles. In contrast, the initial seed generation for the lake *cas* operon had a gap in read coverage of 1.7 Kb between *cas2-cas3* and *csy1* (Figure 3B). The average coverage was higher at 74.4% of the total mappable reads. Two cycles of subset read expansion were necessary to infill the gap between *cas2-cas3* and *csy1* with contiguous read coverage, increasing the average read coverage to 92.4%. With each cycle the average read coverage increased, plateauing at 99.0% with the fifth cycle. The permafrost and wetlands *cas* operons were similar to the lake metagenome with an initial seed generation average read coverage of 75.7 and 88.7%, respectively (Figure 3C and D). After a single cycle of subset read expansion, the average read coverage was 100.0% of the mappable reads for each *cas* operon. While two cycles of subset read expansion had >90% of the mappable reads and infilled a gap of almost 2 Kb between *cas* genes, we set the default parameter for CasCollect to five cycles of subset read expansion to compensate for larger gaps that might exist between *cas* genes in an operon. These additional cycles still permit CasCollect to have massively improved timings compared with the complete assembly.

### Identifying isolated CRISPR arrays

Complete identification of the CRISPR spacers of a target organism is critical for the renewing field of phage therapy. Elucidating CRISPR spacers is necessary for improving the success rate of phage therapy by avoiding the use of phages that the bacterial have immunity by prior CRISPR/Cas system acquisition of complimentary sequences. Many CRISPR arrays are located sufficiently near *cas* genes that these would be revealed by the above method based on Cas protein profiles. However, CRISPR arrays unlinked to *cas* genes are often seen and these arrays would be missed by assembly targeted exclusively to *cas* genes.

To identify these isolated CRISPR arrays, our software includes a DNA mode, primed by CRISPR repeat sequences for the species of interest. CasCollect includes 105 CRISPR repeat sequences (13) as individual Fasta files for CRISPR array detection (Supplemental Table S1). We demonstrate the utility of the DNA mode with a set of genomic sequence data from 66 antibiotic resistant *P. aeruginosa* isolates available from CDC & FDA Antibiotic Resistance Isolate Bank panels (Table 1 and Supplemental Table S2). The data set is especially applicable for being only available in an unassembled raw read format. We used the *P. aeruginosa* CRISPR repeat as input to the CasCollect DNA mode for analyzing each of the CDC isolate datasets to identify seed reads matching *P. aeruginosa* CRISPR arrays. Approximately half the of the strains, 31 of 65, have a *cas* operon with three or more *cas* genes (Table 1). The vast majority of these *cas* operons have an associated CRISPR array, with merely two exceptions. For comparison, the complete assembly pipeline was applied to the same dataset. The two pipelines disagreed for strain AR110 (Table 1, asterisk) for a single CRISPR array with CasCollect yielding 26 spacers and the complete assembly yielding 29 (Supplemental Figure S4A). Interestingly, the three additional CRISPR arrays in the complete assembly appear to be artifacts, perfect copies of one spacer/repeat unit (Supplemental Figure S4B). This was further supported by the lower sequencing coverage specifically for the duplicate CRISPR array region (Supplemental Figure S4C).

**Table 1.** Identification of *P. aeruginosa cas* operons and CRISPR arrays with CasCollect

				CRISPR arrays		CRISPR spacers			Cas genes
Strain	SRA	Reads	Type	Total	Isolated	Total	Isolated	#	Operon
AR054	SRR3112316	18 70 045	TypeIF	4	0	60	0	6	Cas1, Cas3-Cas2, Csy1, Csy2, Csy3, Cas6
			TypeIE					8	Cas2, Cas1, Cas6, Cas5, Cas7, Cse2, Cse1, Cas3
AR064	SRR3290649	12 27 158	TypeIC	1	0	38	0	7	Cas2, Cas1, Cas4, Cas7c, Cas8c, Cas5c, Cas3
AR095	SRR3242025	9 02 598	TypeIE	2	0	48	0	8	Cas3, Cse1, Cse2, Cas7, Cas5, Cas6, Cas1, Cas2
AR103	SRR3112341	24 75 405	TypeIF	4	0	61	0	6	Cas1, Cas3-Cas2, Csy1, Csy2, Csy3, Cas6
			TypeIE					8	Cas2, Cas1, Cas6, Cas5, Cas7, Cse2, Cse1, Cas3
AR108	SRR3112343	20 06 256	TypeIF	3	1	67	14	6	Cas1, Cas3-Cas2, Csy1, Csy2, Csy3, Cas6
AR110	SRR3112345	17 22 889	TypeIF	3	1	64*	15	6	Cas1, Cas3-Cas2, Csy1, Csy2, Csy3, Cas6
AR111	SRR3112346	28 66 888	TypeIF	3	1	66	15	6	Cas1, Cas3-Cas2, Csy1, Csy2, Csy3, Cas6
			TypeU					4	Csf4, Csf1, Csf2, Csf3
AR229	SRR4417530	4 87 104	TypeIF	3	1	37	8	6	Cas1, Cas3-Cas2, Csy1, Csy2, Csy3, Cas6
AR230	SRR4417531	4 74 301	TypeIF	3	1	64	14	6	Cas1, Cas3-Cas2, Csy1, Csy2, Csy3, Cas6
AR235	SRR4417560	6 80 848	TypeIF	2	0	29	0	6	Cas1, Cas3-Cas2, Csy1, Csy2, Csy3, Cas6
AR238	SRR4417563	9 84 680	TypeIE	2	0	18	0	8	Cas2, Cas1, Cas6, Cas5, Cas7, Cse2, Cse1, Cas3
AR241	SRR5122324	16 63 553	TypeU	0	n/a	0	n/a	4	Csf3, Csf2, Csf1, Csf4
AR242	SRR5122326	8 59 269	TypeIF	3	1	65	15	6	Cas1, Cas3-Cas2, Csy1, Csy2, Csy3, Cas6
AR243	SRR5122330	7 20 605	TypeIF	3	1	65	15	6	Cas1, Cas3-Cas2, Csy1, Csy2, Csy3, Cas6
AR245	SRR5122327	11 67 898	TypeU	0	n/a	0	n/a	4	Csf4, Csf1, Csf2, Csf3
AR246	SRR5122329	16 34 253	TypeIF	3	1	36	7	6	Cas1, Cas3-Cas2, Csy1, Csy2, Csy3, Cas6
AR248	SRR5122328	7 98 142	TypeIF	3	1	65	15	6	Cas1, Cas3-Cas2, Csy1, Csy2, Csy3, Cas6
AR249	SRR5122332	22 75 841	TypeIF	3	1	61	16	6	Cas1, Cas3-Cas2, Csy1, Csy2, Csy3, Cas6
AR250	SRR5122323	19 80 810	TypeIF	3	1	36	7	6	Cas1, Cas3-Cas2, Csy1, Csy2, Csy3, Cas6
AR254	SRR4417537	10 00 255	TypeIF	3	1	64	15	6	Cas1, Cas3-Cas2, Csy1, Csy2, Csy3, Cas6
AR255	SRR4417538	8 35 729	TypeIF	3	1	64	15	6	Cas1, Cas3-Cas2, Csy1, Csy2, Csy3, Cas6
AR256	SRR4417539	9 79 368	TypeIF	3	1	40	12	6	Cas1, Cas3-Cas2, Csy1, Csy2, Csy3, Cas6
AR261	SRR4417545	9 14 206	TypeIF	2	0	31	0	6	Cas1, Cas3-Cas2, Csy1, Csy2, Csy3, Cas6
AR262	SRR4417546	10 25 205	TypeIF	4	0	66	0	6	Cas1, Cas3-Cas2, Csy1, Csy2, Csy3, Cas6
			TypeIE					7	Cas2, Cas6, Cas5, Cas7, Cse2, Cse1, Cas3
AR267	SRR4417551	8 30 201	TypeIF	3	1	40	12	6	Cas1, Cas3-Cas2, Csy1, Csy2, Csy3, Cas6
AR268	SRR4417552	9 25 471	TypeIF	4	0	65	0	6	Cas1, Cas3-Cas2, Csy1, Csy2, Csy3, Cas6
			TypeIE					7	Cas2, Cas6, Cas5, Cas7, Cse2, Cse1, Cas3
AR351	SRR6799223	44 31 783	TypeIF	2	0	21	0	6	Cas1, Cas3-Cas2, Csy1, Csy2, Csy3, Cas6
AR355	SRR6799377	42 89 796	TypeIF	3	1	40	5	6	Cas1, Cas3-Cas2, Csy1, Csy2, Csy3, Cas6
AR356	SRR6799380	53 82 136	TypeIF	3	2	26	14	3	Csf2, Cas6, Cas1**
AR358	SRR6799384	32 11 585	TypeIE	3	0	31	0	8	Cas2, Cas1, Cas6, Cas5, Cas7, Cse2, Cse1, Cas3
			TypeIE					6	Cas2, Cas1, Cas6, Cas5, Cas7, Cse2
AR360	SRR6799389	33 30 069	TypeIF	3	1	45	10	6	Cas1, Cas3-Cas2, Csy1, Csy2, Csy3, Cas6

For *cas* operon detection there was a single difference between CasCollect and the complete assembly pipeline. CasCollect identified in strain AR356 a *cas* operon comprising *csf2*, *cas6* and *cas1* (Table 1, double asterisk). This appears to be due to the complete assembly prematurely truncating the end of the contig. Overall, there was very high agreement between CasCollect and the complete assembly for the detection of *cas* operons and CRISPR arrays from unassembled sequencing data of antibiotic resistant patient data. CasCollect improves the performance for detecting *cas* genes and CRISPR arrays and simplifies this process with a single pipeline for inputting raw sequencing data and outputting CRISPR arrays and *cas* genes.

## CONCLUSION

We have developed a software pipeline, CasCollect, for the targeted assembly of CRISPR arrays and *cas* operons. In comparison to the complete assembly, our targeted assembly significantly decreases the time necessary for the identification and annotation of CRISPR/Cas genes from unassembled high-throughput sequencing data by almost two order of magnitude. CasCollect was designed as a flexible platform for read seed collection and read subset expansion prior to assembly; our pipeline includes a set of 120 Cas protein HMMs, yet, can also function with a user-defined set(s) of HMMs for any protein(s) set; also included are 105 CRISPR repeats as reference DNA sequence(s); and a user-defined mode allows for any generated read seed set(s) be used for targeted assembly and CRISPR/Cas annotation. The protein, DNA, or user-defined modes can function as singular searches or can be combined in any combination within a single run. This pipeline was designed for allowing rapid analysis of the multitude of unassembled high-throughput sequencing data that is publicly available and ever increasing. We used a CDC & FDA Antibiotic Resistance Isolate Bank panel of *P. aeruginosa* isolates as a case study for the utility of detecting CRISPR/Cas genes from unassembled sequencing data. While intended for the identification and annotation of CRISPR arrays and *cas* operons, CasCollect can be applied for the targeted assembly of any protein or DNA query.

## DATA AVAILABILITY

CasCollect is publicly available for download under the terms of the GNU General Public License version 3 at in the GitHub repository (https://github.com/sandialabs/CasCollect).

## Supplementary Material

lqaa063_Supplemental_File

## References

[1] Barrangou R., Fremaux C., Deveau H., Richards M., Boyaval P., Moineau S., Romero D.A., Horvath P. CRISPR provides acquired resistance against viruses in prokaryotes. Science. 2007; 315:1709–1712. 17379808 10.1126/science.1138140

[2] Datsenko K.A., Pougach K., Tikhonov A., Wanner B.L., Severinov K., Semenova E. Molecular memory of prior infections activates the CRISPR/Cas adaptive bacterial immunity system. Nat. Commun. 2012; 3:945. 22781758 10.1038/ncomms1937

[3] Makarova K.S., Wolf Y.I., Koonin E.V. Classification and nomenclature of CRISPR-Cas Systems: where from here. CRISPR J. 2018; 1:325–336. 31021272 10.1089/crispr.2018.0033 PMC6636873

[4] Jinek M., Chylinski K., Fonfara I., Hauer M., Doudna J.A., Charpentier E. A programmable dual-RNA-guided DNA endonuclease in adaptive bacterial immunity. Science. 2012; 337:816–821. 22745249 10.1126/science.1225829 PMC6286148

[5] Wagner D.L., Amini L., Wendering D.J., Burkhardt L.M., Akyüz L., Reinke P., Volk H.D., Schmueck-Henneresse M. High prevalence of Streptococcus pyogenes Cas9-reactive T cells within the adult human population. Nat. Med. 2019; 25:242–248. 30374197 10.1038/s41591-018-0204-6

[6] Krylov V.N., Bourkaltseva M.V., Pleteneva E.A. Bacteriophage's Dualism in Therapy. Trends Microbiol. 2019; 27:566–567. 31126841 10.1016/j.tim.2019.05.001

[7] Zhang Q., Doak T.G., Ye Y. Expanding the catalog of cas genes with metagenomes. Nucleic Acids Res. 2014; 42:2448–2459. 24319142 10.1093/nar/gkt1262 PMC3936711

[8] Koonin E.V., Makarova K.S., Zhang F. Diversity, classification and evolution of CRISPR-Cas systems. Curr. Opin. Microbiol. 2017; 37:67–78. 28605718 10.1016/j.mib.2017.05.008 PMC5776717

[9] Makarova K.S., Haft D.H., Barrangou R., Brouns S.J., Charpentier E., Horvath P., Moineau S., Mojica F.J., Wolf Y.I., Yakunin A.F. et al. Evolution and classification of the CRISPR-Cas systems. Nat. Rev. Microbiol. 2011; 9:467–477. 21552286 10.1038/nrmicro2577 PMC3380444

[10] Makarova K.S., Wolf Y.I., Alkhnbashi O.S., Costa F., Shah S.A., Saunders S.J., Barrangou R., Brouns S.J., Charpentier E., Haft D.H. et al. An updated evolutionary classification of CRISPR-Cas systems. Nat. Rev. Microbiol. 2015; 13:722–736. 26411297 10.1038/nrmicro3569 PMC5426118

[11] Shmakov S., Smargon A., Scott D., Cox D., Pyzocha N., Yan W., Abudayyeh O.O., Gootenberg J.S., Makarova K.S., Wolf Y.I. et al. Diversity and evolution of class 2 CRISPR-Cas systems. Nat. Rev. Microbiol. 2017; 15:169–182. 28111461 10.1038/nrmicro.2016.184 PMC5851899

[12] Makarova K.S., Wolf Y.I., Iranzo J., Shmakov S.A., Alkhnbashi O.S., Brouns S.J.J., Charpentier E., Cheng D., Haft D.H., Horvath P. et al. Evolutionary classification of CRISPR–Cas systems: a burst of class 2 and derived variants. Nat. Rev. Microbiol. 2020; 18:67–83. 31857715 10.1038/s41579-019-0299-x PMC8905525

[13] Couvin D., Bernheim A., Toffano-Nioche C., Touchon M., Michalik J., Néron B., Rocha E.P.C., Vergnaud G., Gautheret D., Pourcel C. CRISPRCasFinder, an update of CRISRFinder, includes a portable version, enhanced performance and integrates search for Cas proteins. Nucleic Acids Res. 2018; 46:W246–W251. 29790974 10.1093/nar/gky425 PMC6030898

[14] Grissa I., Vergnaud G., Pourcel C. CRISPRFinder: a web tool to identify clustered regularly interspaced short palindromic repeats. Nucleic Acids Res. 2007; 35:W52–W57. 17537822 10.1093/nar/gkm360 PMC1933234

[15] Biswas A., Staals R.H.J., Morales S.E., Fineran P.C., Brown CM. CRISPRDetect: a flexible algorithm to define CRISPR arrays. BMC Genomics. 2016; 17:356. 27184979 10.1186/s12864-016-2627-0 PMC4869251

[16] Abby S.S., Néron B., Ménager H., Touchon M., Rocha E.P. MacSyFinder: a program to mine genomes for molecular systems with an application to CRISPR-Cas systems. PLoS One. 2014; 9:e110726. 25330359 10.1371/journal.pone.0110726 PMC4201578

[17] Leinonen R., Sugawara H., Shumway M. International Nucleotide Sequence Database Collaboration The sequence read archive. Nucleic Acids Res. 2011; 39:D19–D21. 21062823 10.1093/nar/gkq1019 PMC3013647

[18] Nurk S., Meleshko D., Korobeynikov A., Pevzner P.A. metaSPAdes: a new versatile metagenomic assembler. Genome Res. 2017; 27:824–834. 28298430 10.1101/gr.213959.116 PMC5411777

[19] Langmead B., Salzberg S.L. Fast gapped-read alignment with Bowtie 2. Nat. Methods. 2012; 9:357–359. 22388286 10.1038/nmeth.1923 PMC3322381

[20] Li H., Handsaker B., Wysoker A., Fennell T., Ruan J., Homer N., Marth G., Abecasis G., Durbin R. 1000 Genome Project Data Processing Subgroup The Sequence Alignment/Map format and SAMtools. Bioinformatics. 2009; 25:2078–2079. 19505943 10.1093/bioinformatics/btp352 PMC2723002

[21] Eddy S.R. Accelerated Profile HMM Searches. PLoS Comput. Biol. 2011; 7:e1002195. 22039361 10.1371/journal.pcbi.1002195 PMC3197634

[22] Rognes T., Flouri T., Nichols B., Quince C., Mahé F. VSEARCH: a versatile open source tool for metagenomics. Peer J. 2016; 4:e2584. 27781170 10.7717/peerj.2584 PMC5075697

[23] Bankevich A., Nurk S., Antipov D., Gurevich A.A., Dvorkin M., Kulikov A.S., Lesin V.M., Nikolenko S.I., Pham S., Prjibelski AD. et al. SPAdes: a new genome assembly algorithm and its applications to single-cell sequencing. J. Comput. Biol. 2012; 19:455–477. 22506599 10.1089/cmb.2012.0021 PMC3342519

[24] Bushnell B., Rood J., Singer E. BBMerge - Accurate paired shotgun read merging via overlap. PLoS One. 2017; 12:e0185056. 29073143 10.1371/journal.pone.0185056 PMC5657622

[25] Rho M., Tang H., Ye Y. FragGeneScan: predicting genes in short and error-prone reads. Nucleic Acids Res. 2010; 38:e191. 20805240 10.1093/nar/gkq747 PMC2978382

[26] Ruby J.G., Bellare P., Derisi J.L. PRICE: software for the targeted assembly of components of (Meta) genomic sequence data. G3 (Bethesda, Md.). 2013; 3:865–880. 10.1534/g3.113.005967 PMC3656733 23550143

