# Supplementary material for: CasCollect: targeted assembly of CRISPR-associated operons from high-throughput sequencing data
Source: NAR Genom Bioinform. 2020 Sep 3;2(3):lqaa063. doi: 10.1093/nargab/lqaa063 (PMC7671303; doi:10.1093/nargab/lqaa063)
Supplement: lqaa063_Supplemental_File [file lqaa063_supplemental_file.pdf]

SUPPLEMENTARY TABLE AND FIGURES LEGENDS

Supplementary Figure S1. Comparative analysis of the CasCollect, complete assembly, and PRICE pipelines for isolate genomic datasets. (A) Schematic of the four steps for the CasCollect and complete assembly pipelines: files (blue) for trimming and stripping qualities; seed (orange) for the seed generation, expansion, and reassigning qualities; assembly (grey) for contig building; and annotate (yellow) for detecting CRISPR arrays and *cas* genes GFF3 output file. (B) Timings for each pipeline for each isolate genome and steps. (C) Percentage breakdown for time required for each step. (D) Number of contigs generated by each pipeline with (E) the length distribution for CasCollect, complete assembly, and PRICE. Colors for each step in (A) are used for the charts in (B) and (C).

Supplementary Figure S2. Comparative analysis of the CasCollect and PRICE pipelines for metagenomic datasets. (A) Timings for each pipeline for each isolate genome and steps. (B) Percentage breakdown for time required for each step. The timings for PRICE were stopped after 200 cycles that corresponded to a 3-5 fold time increase compared to CasCollect. The timing for PRICE was insufficient to assemble the majority of *cas* operons.

Supplementary Figure S3. Comparative analysis of the CasCollect and complete assembly pipelines for simulated metagenomic data. (A) Schematic of the four steps for the CasCollect and complete assembly pipelines: Files (blue) for trimming, Seed (orange) for the seed generation by protein mode, Assembly (grey) for contig building, and Annotate (yellow) for the general feature format file of CRISPR/Cas genes. (B) Timings for each pipeline with each step in the pipelines. (C) Percentage breakdown for time required for each step. (D) Number of contig generated by each pipeline with the length distribution for CasCollect (E) and complete assembly (F) characterized. Colors for each step in (A) used for the charts in (B) and (C).

Supplementary Figure S4. Discrepancy in spacer number within CRISPR arrays. (A) Schematic of the CRISPR array from AR110 (SRR3112345) with CasCollect targeted and complete assembly. (B) The sequence of each direct repeat (uppercase) and spacer (lowercase). (C) Read coverage from mapping the raw sequencing reads onto the CRISPR arrays from targeted and complete assembly. Coverage across the targeted assembled was consistent, while the 180 bp region comprising three duplicates of the direct repeat and spacer in the complete assembly had low coverage.

Supplementary Table S1. List of species with CRISPR repeats available for the DNA mode of CasCollect.

Supplementary Table S2. Identification of *Pseudomonas aeruginosa* CRISPR arrays lacking *cas* operons with CasCollect.

Supplemental Fig. S1

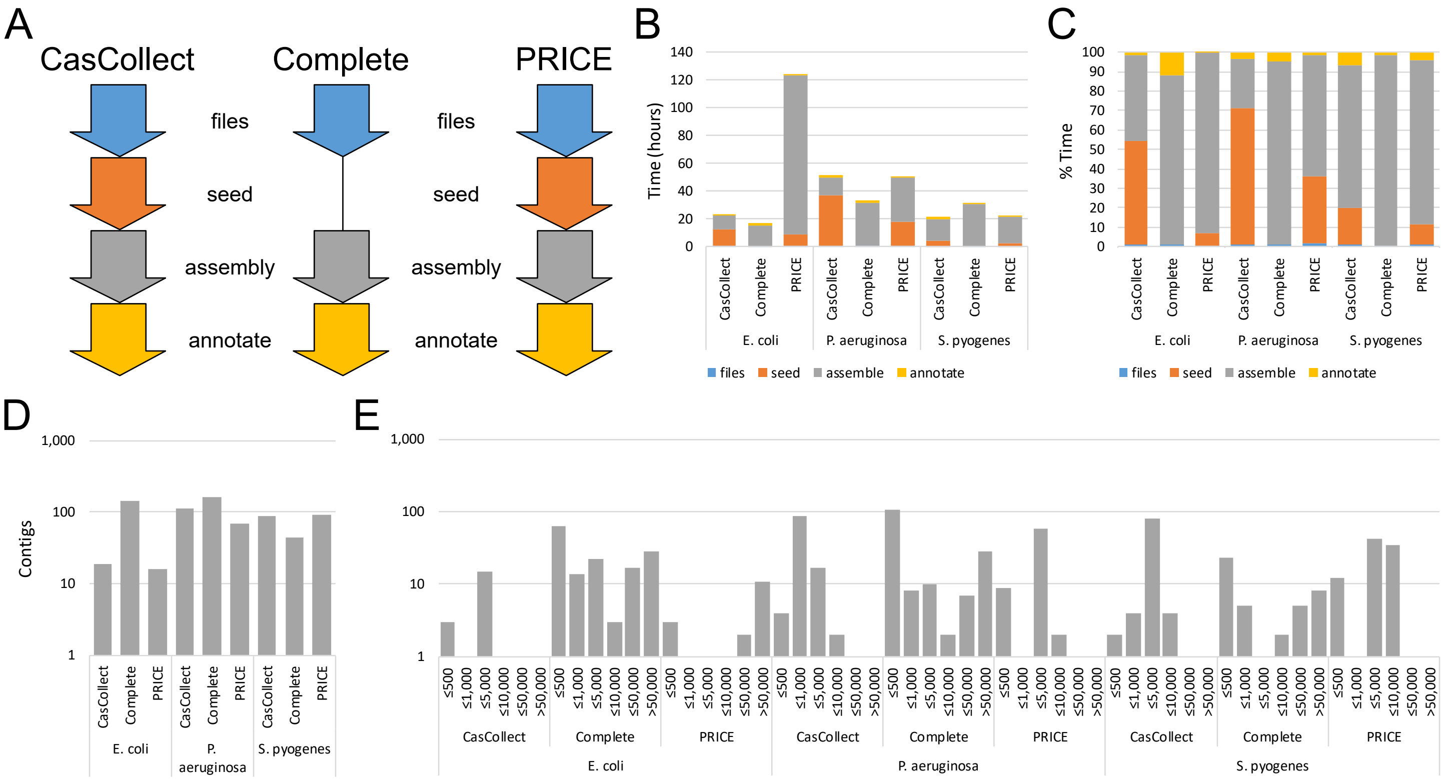

Supplemental Fig. S2

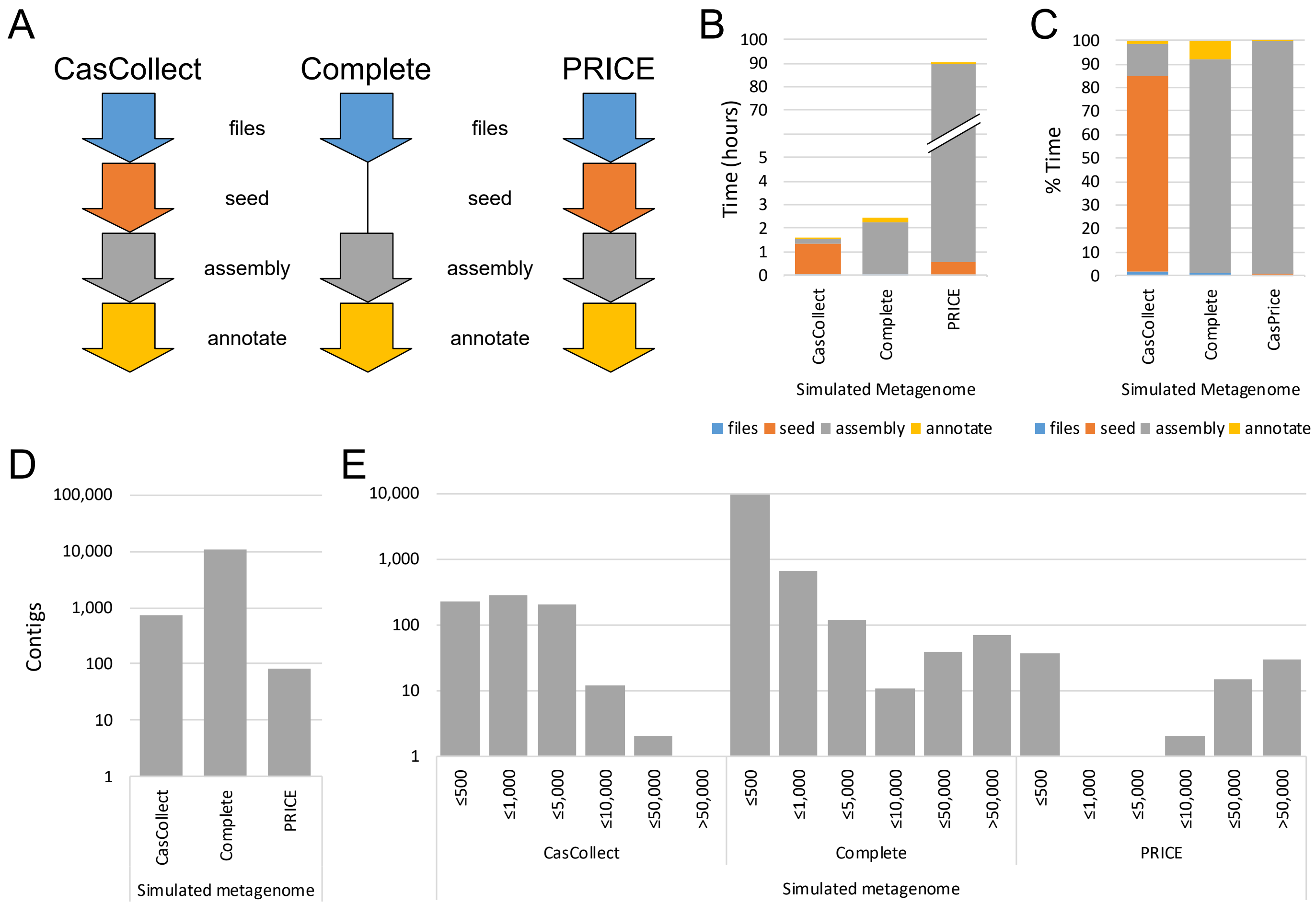

Supplemental Fig. S3

A

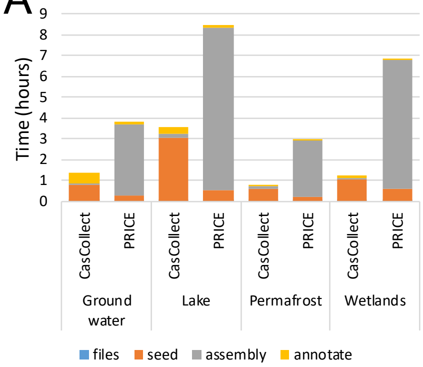

B

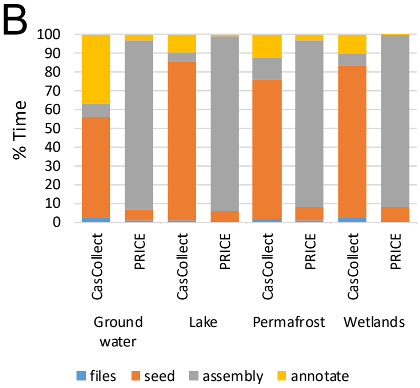

A

AR110 (SRR3112345) assemblies

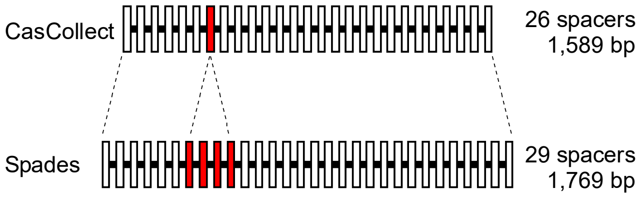

B

```
1                          28                              60
GTTCACTGCCGTGTAGGCAGCTAAGAAAtggctgatcaggctccagaacggatcgtagac
GTTCACTGCCGTGTAGGCAGCTAAGAAAttgatatgccggtagaacgtcgggcgagacat
GTTCACTGCCGTGTAGGCAGCTAAGAAAtcgaacgctgctgagcgccgaacgcatagatg
GTTCACTGCCGTGTAGGCAGCTAAGAAAagaccgaggacggctcgaaaactcggatgatc
GTTCACTGCCGTGTAGGCAGCTAAGAAAttcgacggccacgcctcagcccggcccaggcc
GTTCACTGCCGTGTAGGCAGCTAAGAAAttattgaaatcctcagcggcctgcacttgctc
GTTCACTGCCGTGTAGGCAGCTAAGAAAtgatcacccaagcgtgcgtatcgcggccatcc
GTTCACTGCCGTGTAGGCAGCTAAGAAAtgatcacccaagcgtgcgtatcgcggccatcc
GTTCACTGCCGTGTAGGCAGCTAAGAAAtgatcacccaagcgtgcgtatcgcggccatcc
GTTCACTGCCGTGTAGGCAGCTAAGAAAtgatcacccaagcgtgcgtatcgcggccatcc
GTTCACTGCCGTGTAGGCAGCTAAGAAAtgggtgtccaacatcgacgggtcgaactgctc
GTTCACTGCCGTGTAGGCAGCTAAGAAAtgcgtagatgccgcgatcatagcgcgccctac
GTTCACTGCCGTGTAGGCAGCTAAGAAAttcggtacttctgaaccatacgtcgccgcata
GTTCACTGCCGTGTAGGCAGCTAAGAAAagtcatcgatgaacgacgagccggtcagtgcc
GTTCACTGCCGTGTAGGCAGCTAAGAAAagaagctggagcgacggctggcggcaattcgt
GTTCACTGCCGTGTAGGCAGCTAAGAAAccggacgttcacgctggtggtgagaccatccg
GTTCACTGCCGTGTAGGCAGCTAAGAAAtggctgtcgctgcgctgctggccgctgtgtat
GTTCACTGCCGTGTAGGCAGCTAAGAAAggctggtcccagagcgggtcgacggcacggtc
GTTCACTGCCGTGTAGGCAGCTAAGAAAgaaccgcgcgttcattgctgaaggccatcgtc
GTTCACTGCCGTGTAGGCAGCTAAGAAAacatcagcgccgcggtagccgatgccgatatct
GTTCACTGCCGTGTAGGCAGCTAAGAAAaccatccccggccacgggttgcccgacacctg
GTTCACTGTCGTGTAGGCAGCTAAGAAAgttccatccgggtaggtcacgtccacgtcgta
GTTCACTGCCGTGTAGGCAGCTAAGAAAtggagagtgacccgctcaagaccgaggccgag
GTTCACTGCCGTGTAGGCAGCTAAGAAAtgatgccggacatgggacgtttcgcgggaacc
GTTCACTGCCGTGTAGGCAGCTAAGAAAccggacgcccctaatctggagggctcctggca
GTTCACTGCCGTGTAGGCAGCTAAGAAAtttccccgcgaggcatagcagggatatcttgt
GTTCACTGCCGTGTAGGCAGCTAAGAAAtacaagcagatcggcgagctgagcggcaagga
GTTCACTGCCGTGTAGGCAGCTAAGAAAtcgtactggtcgagatttccgattcggaagcc
GTTCACTGCCGTGTAGGCAGCTAAGAAAttcgacgccgctagggttgccgcatcgccgtc
GTTCACTGCCGTGTAGGCAGCTAAGAAA
```

C

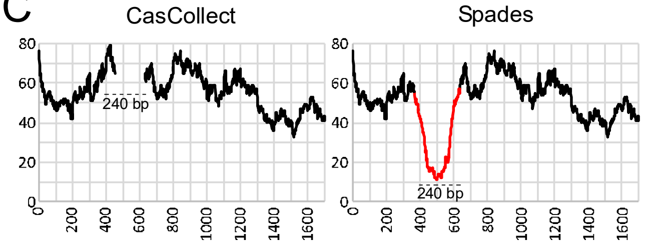

CRISPR array repeats	
Aeropyrum camini	Microlunatus phosphovorus NM-1
Alicyclobacillus acidocaldarius subsp acidocaldarius Tc-4-1	Mycobacterium canettii CIPT 140010059
Azotobacter vinelandii CA	Mycobacterium tuberculosis RGTB327
Bdellovibrio exovorus JSS	Mycoplasma gallisepticum S6
Bibersteinia trehalosi USDA-ARS-USMARC-192	Myxococcus stipitatus DSM 14675
Bifidobacterium longum subsp longum KACC 91563	Neisseria meningitidis Z2491
Brachyspira hyodysenteriae WA1	Nocardia cyriacigeorgica GUH-2
Burkholderia sp YI23	Nocardiopsis alba ATCC BAA-2165
Calothrix sp PCC 6303	Nostoc sp PCC 7120
Campylobacter sp 03-427	Pasteurella multocida 36950
Candidatus Arthromitus sp SFB-mouse-Japan	Pectobacterium carotovorum subsp carotovorum PCC21
Chamaesiphon minutus PCC 6605	Persephonella marina EX-H1
Clostridium sp SY8519	Phycisphaera mikurensis NBRC 102666
Corynebacterium terpenotabidum Y-11	Pleurocapsa sp PCC 7327
Crinalium epipsammum PCC 9333	Porphyromonas gingivalis TDC60
Cronobacter sakazakii ES15	Propionibacterium acidipropionici ATCC 4875
Cycloclasticus zancles 7-ME	Providencia stuartii MRSN 2154
Dehalococcoides mccartyi DCMB5	Pseudomonas aeruginosa DK2
Desulfocapsa sulfexigens DSM 10523	Pyrobaculum sp 1860
Desulfovibrio hydrothermalis AM13	Pyrococcus sp ST04
Enterococcus faecalis D32	Ralstonia solanacearum Po82
Escherichia coli str K-12 substr MDS42 DNA	Rhodospirillum photometricum DSM 122
Fervidicoccus fontis Kam940	Rhodospirillum rubrum F11
Flavobacterium branchiophilum	Riemerella anatipestifer RA-CH-1
Frankia sp CcI3	Saccharothrix espanaensis DSM 44229
Gallibacterium anatis UMN179	Salinispora arenicola CNS-205
Geitlerinema sp PCC 7407	Salmonella enterica subsp enterica serovar Typhimurium str 798
Geobacillus thermoleovorans CCB US3 UF5	Sorangium cellulosum So0157-2
Gloeobacter sp JS	Spiroplasma apis B31
Gloeocapsa sp PCC 7428	Stanieria cyanosphaera PCC 7437
Gordonia polyisoprenivorans VH2	Staphylococcus aureus 08BA02176
Granulibacter bethesdensis CGDNIH1	Streptococcus equi subsp zooepidemicus ATCC 35246
Halorhabdus tiamatea SARL4B	Streptococcus lutetiensis 033
Helicobacter cinaedi PAGU611	Streptococcus macedonicus ACA-DC 198
Hydrogenobaculum sp 3684	Streptococcus salivarius CCHSS3
Hyphomicrobium nitrativorans NL23	Streptococcus suis YB51
Kibdelosporangium phytohabitans strain KLBMP1111	Streptococcus thermophilus JIM 8232
Klebsiella oxytoca KCTC 1686	Streptomyces hygroscopicus subsp jinggangensis 5008
Lactobacillus brevis KB290 DNA	Sulfobacillus acidophilus TPY
Lactobacillus ruminis ATCC 27782	Sulfolobus acidocaldarius N8
Leptothrix cholodnii SP-6	Synechococcus sp PCC 7502
Leuconostoc gelidum JB7	Synechocystis sp PCC 6803
Listeria ivanovii subsp londoniensis strain WSLC 30167	Taylorella asinigenitalis MCE3
Listeria monocytogenes M7	Tepidanaerobacter acetatoxydans Re1
Mannheimia haemolytica USDA-ARS-SAM-185	Thermacetogenium phaeum DSM 12270
Megasphaera elsdenii DSM 20460	Thermoanaerobacterium saccharolyticum JW-SL-YS485
Melioribacter roseus P3M	Thermococcus sp 4557
Melissococcus plutonius DAT561	Thermogladius sp 1633
Metallosphaera cuprina Ar-4	Thermotoga elfii NBRC 107921
Methanocella sp HZ254	Thermus sp CCB US3 UF1
Methanoculleus bourgensis MS2T	Thioalkalivibrio nitratireducens DSM 14787
Methanosaeta harundinacea 6Ac	Tistrella mobilis KA081020-06
Microcoleus sp PCC 7113	

Strain	SRA	Reads	Type	CRISPR		Cas Genes	
				Arrays	Spacers	#	Operon
AR090	SRR3112335	2,126,644	n/a	0	0	0	n/a
AR092	SRR3112336	1,225,103	n/a	0	0	0	n/a
AR094	SRR3112337	1,657,533	n/a	0	0	0	n/a
AR100	SRR3112340	2,087,105	n/a	0	0	0	n/a
AR105	SRR3112342	1,989,806	n/a	0	0	0	n/a
AR231	SRR4417542	478,617	n/a	1	8	0	n/a
AR232	SRR4417553	375,904	n/a	0	0	0	n/a
AR233	SRR4417558	615,859	n/a	0	0	0	n/a
AR234	SRR4417559	477,110	n/a	0	0	0	n/a
AR236	SRR4417561	488,238	n/a	0	0	0	n/a
AR237	SRR4417562	1,175,855	n/a	0	0	0	n/a
AR239	SRR4417532	1,008,367	n/a	1	4	0	n/a
AR240	SRR5122331	1,647,468	n/a	0	0	0	n/a
AR244	SRR4417533	856,508	n/a	0	0	0	n/a
AR247	SRR4417534	856,694	n/a	0	0	0	n/a
AR251	SRR4417535	882,484	n/a	0	0	0	n/a
AR252	SRR5122325	1,141,358	n/a	0	0	0	n/a
AR253	SRR4417536	884,811	n/a	0	0	0	n/a
AR257	SRR4417540	951,684	n/a	0	0	0	n/a
AR258	SRR4417541	975,106	n/a	0	0	0	n/a
AR259	SRR4417543	881,366	n/a	0	0	0	n/a
AR260	SRR4417544	953,363	n/a	1	12	0	n/a
AR263	SRR4417547	918,300	n/a	0	0	0	n/a
AR264	SRR4417548	877,917	n/a	1	10	0	n/a
AR265	SRR4417549	766,911	n/a	1	13	0	n/a
AR266	SRR4417550	857,564	n/a	0	0	0	n/a
AR269	SRR4417554	1,011,282	n/a	0	0	0	n/a
AR270	SRR4417555	1,012,411	n/a	0	0	0	n/a
AR271	SRR4417556	896,578	n/a	0	0	0	n/a
AR272	SRR4417557	777,887	n/a	0	0	0	n/a
AR352	SRR6985662	7,982,657	n/a	1	14	0	n/a
AR353	SRR6799371	3,957,778	n/a	1	10	0	n/a
AR354	SRR6799376	4,153,625	n/a	0	0	0	n/a
AR357	SRR6799382	4,035,637	n/a	0	0	0	n/a
AR359	SRR6799387	3,194,525	n/a	0	0	0	n/a
